# Psychometric properties of the post-stroke depression scale in the sequelae stage

**DOI:** 10.3389/fpsyg.2023.1130497

**Published:** 2023-03-31

**Authors:** Yawei Zeng, Fengzhen Li, Liuqiao Ning, Yingjie Fu, Yajing Ge, Beibei Gan, Suichai Lin, Haiyun Lin, Jufang Li

**Affiliations:** ^1^School of Nursing, Wenzhou Medical University, Wenzhou, China; ^2^Department of Wound Repair, First Affiliated Hospital of Wenzhou Medical University, Wenzhou, China; ^3^Department of Respiratory and Critical Care Medicine, First Affiliated Hospital of Wenzhou Medical University, Wenzhou, China; ^4^Department of Emergency, First Affiliated Hospital of Wenzhou Medical University, Wenzhou, Zhejiang, China; ^5^Department of Rehabilitation Medicine, First Affiliated Hospital of Wenzhou Medical University, Wenzhou, Zhejiang, China

**Keywords:** post-stroke depression, exploratory factor analysis, confirmatory factor analysis, sequelae stage, measurement development

## Abstract

**Aim:**

To evaluate the psychometric properties of the Post-Stroke Depression Scale in the Sequelae Stage (PSDS-SS).

**Background:**

The incidence of the sequelae stage Post-Stroke Depression (PSD) is high, and the best screening tools are still lacking. Under this circumstances, our research team developed the PSDS-SS by Delphi method, but its psychometric properties need to be further verified.

**Method:**

This was a cross-sectional study. Seven hundred and sixteen stroke patients in the sequelae stage were enrolled by purpose sampling from May 2022 to September 2022. The exploratory factor analysis (EFA) and confirmatory factor analysis (CFA) were used to verify the factor structure of the scale. The reliability of the scale was tested by Cronbach’s *α* coefficient, test–retest reliability and composite reliability. The validity of the scale was tested by criterion-related validity, convergent and discriminant validity.

**Result:**

Eight items were deleted through item analysis. The EFA ended up with a 5-factor scale including 24 items after removing one item with low factor loading. Finally, a 21-item model was established by confirmatory factor analysis, and all the fit indexes were acceptable. The reliability and validity of the total scale and each factor are acceptable.

**Conclusion:**

The PSDS-SS has a stable factor structure, and demonstrated good reliability and validity. And it would be an effective tool to assess PSD in the sequelae stage.

## 1. Introduction

Post stroke depression (PSD) is a common neuropsychiatric complication after stroke (Paolucci, 2017; Camargos et al., 2020). As a chronic recurrent disease, the incidence of PSD is about one-third in different periods of stroke (Hackett et al., 2014; Paolucci, 2017). PSD occurs in the early stage, rehabilitation stage and sequelae stage after stroke. The symptoms of PSD in these three different stages are quite different (Li et al., 2019). PSD in the sequelae stage refers to depression occurring 6 months after stroke (Wang, 2009; Zhang et al., 2022). The main symptoms of PSD in the sequelae stage are insomnia, more irritability than usual, easy to be fatigued, decreased sensory ability, loss of speech, suicidal ideation and easy to be crying (Li et al., 2019). PSD in the sequelae stage can seriously affect patients’ functional recovery, rehabilitation outcome and quality of life. Firstly, PSD in sequelae stage significantly worsens the physical function, affects the prognosis of patients, and eventually increases the mortality and disability rate (Maaijwee et al., 2016; Pan et al., 2016; McCurley et al., 2019; Virani et al., 2020). Secondly, it can also reduce the patients’ desire for active rehabilitation, thus affecting the rehabilitation effect of language, movement, walking and other aspects (Huang and Wang, 2016). Finally, PSD would make it difficult for patients to return to work and reduce their quality of life (Mutai et al., 2016; Stein et al., 2018).

**Figure 1:**
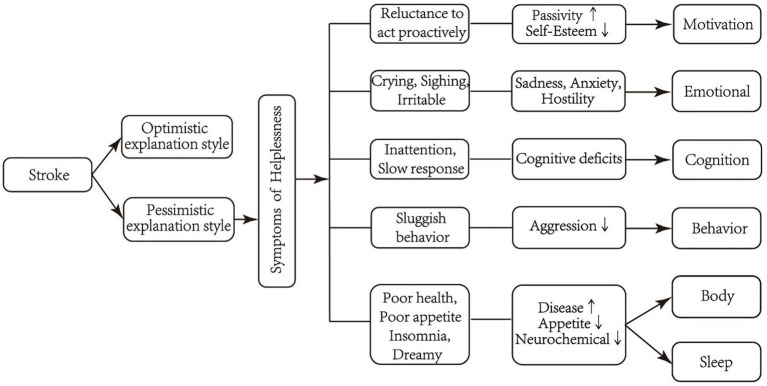
Conceptual framework.

Despite the high prevalence and adverse effects of PSD in the sequelae stage, it has not been effectively screened and managed (Lanctôt et al., 2020). The lack of specific screening tools maybe the main reason for this phenomenon. Thus, we developed the Post-Stroke Depression Scale in the Sequelae Stage (PSDS-SS) with 8 dimensions and 33 items through literature review, semi-structured interviews with PSD patients in the sequelae stage, research group discussion, healthcare professional panel evaluation and Delphi consultation. Notably, the theory of learned helplessness was used as the theoretical basis for scale development. Learned helplessness refers to the cognitive, motivational, and emotional deficits when people experiencing an event beyond their control (Alloy et al., 1984; Peterson and Seligman, 1984). The manifestation of learned helplessness is similar to depression. Thus, learned helplessness theory is one of the important theories to explain depression (Wang and Zhang, 2004). Based on the theory of learned helplessness, we built up the conceptual framework of this study as a guidance of scale development (Figure 1). In the conceptual framework, stroke was supposed to be a stimulating event, which incurred six factors of PSD symptoms, namely, motivation, emotion, cognition, behavior, somatization and sleep. And the item pool of the scale were constructed based on the six. The aim of this study was to evaluate the psychometric properties of the PSDS-SS, so as to provide a specific tool for the screening of the sequelae stage PSD.

## 2. Background

At present, the diagnostic and screening tools for the sequelae stage PSD include mental disease diagnostic systems, ordinary depression rating scales and specific PSD screening scale. But the above tools all have some limitations.

### 2.1. Mental disease diagnostic systems

There are three mental disease diagnostic systems used to diagnose the sequelae stage PSD: The diagnostic and statistical manual of mental disorders-V (DSM-V); International Classification of Diseases-10 (ICD-10); Chinese Classification and Diagnostic Criteria of Mental Disorders-3 (CCMD-3). The mental disease diagnostic systems used to screen for the sequelae stage PSD have the following problems: (1) Due to the different causes and symptoms of PSD and functional depression, DSM-V, ICD-10, and CCMD-3 do not specify the concept and classification of PSD. So the classification and diagnosis of functional depression are not applicable to PSD (Gainotti et al., 1997; Ma et al., 2021). (2) The need for diagnosis by a physician limits its use in nursing staff (Li et al., 2017). (3) The DSM-V is the standard for diagnosing severe functional depression, which make patients miss the best opportunity for non-pharmacological management.

### 2.2. Ordinary depression rating scale

The ordinary depression rating scales for the sequelae stage PSD can be divided into examiner-rating scale and self-rating scale. Examiner-rating scales mainly include: Hamilton depression Rating Scale (HAMD), Montgomery and Asberg Depression Rating Scale (MADRS). Self-rating scales mainly include: Beck Depression Inventory-II (BDI-II), Patient Health Questionnaire-9 (PHQ-9), Zung Self-rating Depression Scale (SDS), Center for Epidemiological Studies Depression Scale (CES-D), Hospital Anxiety and Depression Scale (HADS), Geriatric Depression Scale (GDS). However, the above scales are not the best tools for screening PSD in sequelae stage for the following reasons: (1) These scales are not designed for PSD and therefore lack specific items for PSD, resulting in a lack of specificity in screening results (de Coster et al., 2005; Kang et al., 2013; Leng et al., 2020). (2) Some scales need to be evaluated by professional raters, which limits their widespread use. (3) The reliability and validity of some scales in the measurement of the sequelae stage PSD need to be further verified (Roger and Johnson-Greene, 2009; Adams, 2011; Prisnie et al., 2016).

### 2.3. Specific PSD screening scale

The specific PSD screening scales used to screen PSD in the sequelae stage mainly include: The Post-Stroke Depression Rating Scale (PSDRS), Post-Stroke Depression Scale (PSDS). These scales has the following limitations for screening PSD in the sequelae stage: (1) The PSDRS scale cannot evaluate and classify the severity of PSD (Gainotti et al., 1997), and the screening results are greatly affected by age (Quaranta et al., 2008). In addition, its accuracy in the assessment of sequelae stage PSD is insufficient (Quaranta et al., 2008). (2) The reliability and validity of PSDS has not been verified in large samples, and whether its screening results are affected by other factors needs to be further tested (Yue et al., 2015).

In summary, due to the lack of specific screening tools for sequelae stage PSD at present, our research team developed a specific screening scale for the sequelae stage PSD by Delphi method based on the theory of learned helplessness. This study aimed to evaluate the psychometric properties of the PSDS-SS.

## 3. Methods

### 3.1. Participants

Seven hundred and sixteen stroke patients in the sequelae stage were enrolled by purpose sampling from March 2022 to September 2022 in a Grade A class 3 hospital in southeast China. The inclusion criteria were patients: (1) who met the diagnostic criteria for stroke; (2) whose vital signs were stable after stroke; (3) who were ill for more than 6 months, that is, in the sequelae stage of stroke; (4) who were able to communicate in language; (5) who gave informed consent. The exclusion criteria were patients: (1) who were suffering from subarachnoid hemorrhage; (2) with severe heart, liver, renal insufficiency, respiratory failure, malignant tumor and other serious diseases.

The sample size was determined according to the requirements of exploratory factor analysis (EFA) and confirmatory factor analysis (CFA). EFA and CFA use two data sets independently. EFA requires that the number of participants should be 5 to 10 times the number of scale items (Ding et al., 2022). The PSDS-SS obtained by Delphi method has 33 items, and the required sample size should be 165–330. Considering the non-response bias, the sample size should be increased by 10% and the required sample size should be 182–363. A sample size of 350 was assumed for EFA. In addition, the sample size of the CFA needs to be larger than that of the EFA (Li et al., 2019). Thus, we assumed a sample of more than 350 for the CFA. Finally, 716 patients were enrolled in the study. They were randomly divided into EFA and CFA data sets, including 356 patients in EFA and 360 patients in CFA. Forty-five participants were selected to fill in the PHQ-9 scale to test the criterion-related validity. In addition, 40 samples were selected to fill in the PSDS-SS again after 2 weeks to evaluate the test–retest reliability.

### 3.2. Measure

#### 3.2.1. General demographic questionnaire

The general demographic questionnaire included the following items: (1) general information of patients: age, sex, educational level, marital status, with child or not, ethnicity, religion and personal income after stroke; (2) disease information of the patients: months after stroke, number of strokes, stroke type, stroke site, presence of comorbidities (hypertension, diabetes, dyslipidemia, heart disease and coronary heart disease).

#### 3.2.2. The post-stroke depression scale in the sequelae stage

The PSDS-SS was developed by our research team. It was initially constructed through literature review, semi-structured interviews with PSD patients in the sequelae stage, research group discussion and healthcare professional panel evaluation. Then, a two-round Delphi consultation was conducted. For the two-round Delphi consultation, 18 experts were responded in the first round, and 15 experts were responded in the second round. Their areas of expertise include applied psychology, psychiatric nursing, community and aged care, stroke nursing, stroke rehabilitation and depression treatment. There are 4 nurses, 3 doctors, 10 nursing teachers and 1 psychological worker in the first round, with a working experience of 13–40 years. There are 4 nurses, 2 doctors, 8 nursing teachers and 1 psychological worker in the second round, with a working experience of 13–40 years. The positive coefficient of the two-round Delphi consultation were 81.81 and 83.33%. The authority coefficient of the two-rounds Delphi were 0.90 and 0.89. The Kendall’s W of two-rounds Delphi were 0.135 (*p* < 0.01) and 0.223 (*p* < 0.01). The means for the two rounds ranged from 3.83 to 4.89 and 3.93 to 5.00 respectively; the full score ratios ranged from 0.28 to 0.89 and 0.47 to 1.00 respectively; the coefficient of variation ranged from 0.07 to 0.36 and 0.00 to 0.33, respectively. The item-level content validity index (I-CVI) ranged from 0.56 to 1.00 and 0.67 to 1.00 in the first and second rounds consultation. The inclusion criteria of items were mean ≥ 4.00, CV ≤ 0.20, full score ratio ≥ 0.50 and I-CVI ≥ 0.78. After removing items that did not meet the inclusion criteria, we obtained the PSDS-SS with 8 factors (motivation, emotion, cognition, behavior, somatization, sleep, helplessness, and guilt) and 33 items. All items of the PSDS-SS had an I-CVI >0.78, and the S -CVI/Ave was 0.941. The PSDS-SS used a 5-point Likert scale ranging from 1 to 5, corresponding to “none,” “occasionally,” “sometimes,” “often” and “always.” The total score of scale was the sum of all items, and the score of factor was the sum of items in each factor. The total score of scale was 33–165 points in total. A higher score indicated more severe depressive symptoms.

#### 3.2.3. PHQ-9

PHQ was developed by Spitzer et al. in 1999 (Spitzer et al., 1999). PHQ-9 was localized in 2009 (Bian et al., 2009) to measure depression in participants who attending medical, gynecologic, and surgical outpatient department. ThePHQ-9 uses a 4-point Likert scale ranging from 0 to 3, corresponding to “none,” “few days,” “more than half” and “almost everyday” When PHQ-9 is used for screening PSD, the cut-off value is mostly ≥5 points (Yang, 2016). The Cronbach’s *α* of PHQ-9 was 0.78 in the stroke patients (Dajpratham et al., 2020).

### 3.3. Data collection

Patients who met the inclusion criteria were selected as potential participants through the electronic medical record system. Data were collected by one-to-one telephone interviews. After informed consent was obtained, the researcher read out the items of the general demographic questionnaire, the PSDS-SS and PHQ-9 to the participants and recorded their answers. Each interview took about 20 min to complete.

### 3.4. Data analysis

SPSS 24.0 was used for descriptive analysis (mean, standard deviation, frequency and percentage), item analysis and EFA. Amos 24.0 was used for CFA.

#### 3.4.1. Item analysis

Item analysis includes critical ratio (CR), correlation analysis, Cronbach’s *α* test and factor analysis. Items are deleted when they do not meet two or more of the following criteria: (1) the CR was significant, and should be >3; (2) the correlation between item scores and total scores, and should be significant and >0.40; (3) the Cronbach’s *α* after deleting an item is smaller than the Cronbach’s *α* of the total scale before deleting it; (4) factor loading of the items were >0.45; (5) item commonalities were >0.20 (Liu et al., 2022).

#### 3.4.2. EFA

The Kaiser-Meyer-Olkin (KMO) index and Bartlett’s test of sphericity were used to evaluated whether the sample was suitable for EFA. If the Bartlett’s test of sphericity is significant and KMO value is >0.60, the scale is suitable for EFA (Wynter et al., 2017; Forsell et al., 2019). In EFA, Kaiser eigenvalue >1 and scree plot were used to select factors. It is also required that the factor loading of the reserved item should be >0.45 (Şenyuva et al., 2020).

#### 3.4.3. CFA

CFA used maximum likelihood method to estimate the parameters. Because of the violation of normal distribution and multivariate normality, the Bollen–Stine bootstraps were used to estimate chi-square as a model fit index (Komlenac et al., 2018). Three types of model fit indexes were employed to determine the model fit: absolute indexes, relative indexes and parsimony indexes. The absolute index included chi-square value/degrees of freedom (*χ*^2^/df), Standardized root mean square residual (SRMR), Root-mean-square error of approximation (RMSEA), Goodness-of-fit index (GFI) and Adjusted goodness-of-fit index (AGFI). It is considered acceptable that *χ*^2^/df < 5.00, SRMR <0.08, RMSEA ≤0.08, GFI and AGFI >0.80 (Hooper et al., 2008; Haigh et al., 2011; Duan et al., 2015; Huang et al., 2022). The relative indexes included Tueker-Lewisindex (TLI) and Comparative fit index (CFI). When TLI and CFI are >0.90, the model is considered to have a good fit, but some studies believe that TLI and CFI are also acceptable when them are closer to 0.90 (Brust et al., 2022). Parsimony-adjusted normed fit index (PNFI) and Parsimony comparative fit index (PCFI) were selected as parsimony indexes. The values PNFI and PCFI >0.50 indicates that the model is acceptable (Feizi and Heidari, 2020).

#### 3.4.4. Criterion-related validity, convergent and discriminant validity

The correlation coefficient between PHQ-9 and The PSDS-SS was used to reflect the criterion-related validity. Pearson correlation was used for normal data, and Spearman correlation was used for non-normal data. A significant correlation ranged from 0.40 to 0.80 indicated good criterion-related validity of the scale. The convergent validity was represented by the Average Variance Extracted (AVE), which should be >0.50 (Liu et al., 2022). The discriminant validity is illustrated by comparing the square root of AVE of each factor with the correlation coefficient of this factor and any other factor. The square root of AVE larger than the correlation coefficient between each factor and other factors indicates that the discriminant validity is good (Mo-xi and Xin, 2019).

#### 3.4.5. Reliability

The Cronbach’ *α*, Test–retest reliability and Composite reliability (CR) were used to evaluate the reliability of the scale. Cronbach’ *α* was used to evaluate the internal consistency reliability, which needed to be >0.70 (Liu et al., 2022). The retest reliability was calculated by Intraclass correlation coefficient (ICC). When ICC < 0.40, the scale reliability is poor. 0.40 ≤ ICC < 0.75 indicates acceptable reliability; ICC ≥ 0.75 indicates good reliability (Padilha et al., 2020). CR > 0.70 is considered to have an acceptable composite reliability (Hu et al., 2021).

### 3.5. Ethical considerations

This study was approved by the Hospital Ethics Committee (approval number: KY2022-070). The researchers explained the purpose and significance of the study to the participants before data collection. The patients provided informed consent in this study. The data of patients were kept confidential and were not accessible to any person other than the research team members.

## 4. Results

### 4.1. General demographic of the participants

This study finally obtained 716 valid samples, which were randomly divided into two datasets to run EFA (*N* = 356) and CFA (*N* = 360). The demographic information is shown in Table 1, and there were no differences in demographic characteristics between the EFA and CFA samples.

**Table 1:** General demographic questionnaire.

Characteristic	EFA (*N* = 356)	CFA (*N* = 360)	Statistic	Value of *p*
Age	61.671 (11.151，29–92)	61.042 (11.151，27–87)	*Z* = −0.870	0.384
Sex
Male	231 (64.9)	244 (67.8)	*χ*^2^ = 0.670	0.413
Female	125 (35.1)	116 (32.2)		
Educational status
Illiteracy	82 (23.0)	81 (22.5)	*χ*^2^ = 2.795	0.593
Primary school	142 (39.9)	160 (44.4)		
Junior high school	88 (24.7)	72 (20.0)		
High school	31 (8.7)	34 (9.4)		
University or above	13 (3.7)	13 (3.6)		
Marital status
Married	336 (94.4)	335 (93.1)	*F* = 1.397	0.740
Unmarried	8 (2.2)	7 (1.9)		
Divorced	2 (0.6)	4 (1.1)		
Widowed	10 (2.8)	14 (3.9)		
Children
Have children	345 (96.9)	348 (96.7)	*χ*^2^ = 0.034	0.853
No children	11 (3.1)	12 (3.3)		
Nation
Han	348 (97.8)	356 (98.9)	*χ*^2^ = 1.402	0.236
Minority	8 (2.2)	4 (1.1)		
Religion
Have	132 (37.1)	113 (31.4)	*F* = 3.607	0.106
Not	223 (62.6)	247 (68.6)		
Income after stroke
Salary or pension	132 (37.1)	157 (43.6)	*F* = 4.661	0.157
Security fund	28 (7.9)	21 (5.8)		
Family support	196 (55.1)	181 (50.3)		
No fixed income	0 (0)	1 (0.3)		
Months after stroke	8.406 (7.691)	7.723 (4.832)	Z = −0.032	0.974
Stroke frequency	1.132 (0.406)	1.144 (0.491)	*Z* = −0.051	0.959
Stroke type
Hemorrhagic stroke	63 (17.7)	53 (14.7)	*χ*^2^ = 4.586	0.105
Ischemic stroke	278 (78.1)	300 (83.3)		
Lacunar infarction	15 (4.2)	7 (1.9)		
Stroke site
Left hemisphere	151 (42.4)	143 (39.7)	*χ*^2^ = 1.098	0.783
Right hemisphere	126 (35.4)	136 (37.8)		
Cerebellum or brain stem	64 (18.0)	69 (19.2)		
Multiple site	15 (4.2)	12 (3.3)		

### 4.2. Item analysis

The CR for all items were >3.00 (*p* < 0.001). The correlation coefficient between items 3, 10, 11, 12, 19, 20, 22, 23, and 25 and the total score was <0.40. The Cronbach’s *α* for the total scale was 0.927, and the Cronbach’s *α* for the scale increased after the removal of items 10, 16, 19, and 23. In factor analysis, the commonality of items 10, 12, 16, 19, 20, 21, 22, 23 was <0.20, and the factor load was <0.45. Items 10, 12, 16, 19, 20, 21, 22, and 23 that did not meet two or more of the criteria which were considered to be deleted in this step. Finally, 25 items were retained (Table 2).

**Table 2:** The result of item analysis (*N* = 356).

Item	CR ≥ 3	Correlation between item and total score	Cronbach ‘α after item deletion	Factor analysis		The number of criteria not met	Items retained
				Commonality	Factor loading		
1. I do not want to take the initiative to do things	8.296**	0.464**	0.926	0.205	0.453	0	Selected
2. I do not want to go out	7.986**	0.463**	0.926	0.212	0.460	0	Selected
3. I do not want to communicate with people	6.598**	0.397**	0.926	0.252	0.502	1	Selected
4. I lost interest in things that I used to be interested in	8.825**	0.511**	0.925	0.292	0.540	0	Selected
5. I feel down	9.317**	0.545**	0.925	0.374	0.611	0	Selected
6. I feel hopeless	7.963**	0.539**	0.924	0.537	0.732	0	Selected
7. I feel anxious	8.593**	0.561**	0.924	0.442	0.665	0	Selected
8. I worry about the future	9.114**	0.574**	0.924	0.398	0.631	0	Selected
9. I can hardly be happy	8.256**	0.521**	0.925	0.307	0.554	0	Selected
10. My memory has deteriorated	5.462**	0.326**	0.929	0.064	0.253	4	Deleted
11. My ability to think about problems has declined	5.879**	0.388**	0.926	0.213	0.462	1	Selected
12. My mind has become dull	4.998**	0.313**	0.927	0.134	0.366	3	Deleted
13. I have fewer social activities	9.969**	0.564**	0.925	0.333	0.577	0	Selected
14. I cry or want to cry	6.490**	0.450**	0.925	0.387	0.622	0	Selected
15. I think a lot	9.411**	0.584**	0.924	0.406	0.637	0	Selected
16. I feel tired	6.969**	0.441**	0.928	0.121	0.349	3	Deleted
17. I find it difficult to manage everyday life	8.876**	0.550**	0.924	0.460	0.679	0	Selected
18. I am not satisfied with my current physical condition	10.590**	0.644**	0.923	0.483	0.695	0	Selected
19. I woke up in the middle of the night	6.976**	0.397**	0.928	0.085	0.291	4	Deleted
20, I do not fall asleep easily	5.857**	0.337**	0.927	0.151	0.388	3	Deleted
21. I have trouble falling asleep after waking up in the middle of the night	7.172**	0.415**	0.926	0.163	0.403	2	Deleted
22. I get less sleep	6.179**	0.343**	0.926	0.153	0.391	3	Deleted
23. I dream a lot while I sleep	4.782**	0.277**	0.928	0.074	0.272	4	Deleted
24. I think the recovery effect is much lower than I expected	8.820**	0.577**	0.924	0.464	0.681	0	Selected
25. I do not think friends and family fully understand me	5.536**	0.377**	0.926	0.298	0.546	1	Selected
26. I do not feel like my body is getting better	11.289**	0.710**	0.922	0.649	0.806	0	Selected
27. I feel powerless about a lot of things	11.471**	0.746**	0.922	0.604	0.777	0	Selected
28. I feel isolated	6.418**	0.445**	0.925	0.420	0.648	0	Selected
29. I do not think I can change my current status	10.988**	0.695**	0.922	0.571	0.756		Selected
30. I think I’m so useless	10.302**	0.635**	0.923	0.508	0.713	0	Selected
31. I consider myself a burden on the family	9.537**	0.558**	0.924	0.400	0.632	0	Selected
32. I feel guilty for dragging down my family	9.206**	0.555**	0.924	0.411	0.641	0	Selected
33. I do not want to live	5.696**	0.427**	0.926	0.415	0.644	0	Selected

**p < 0.001; *p < 0.05.

### 4.3. EFA

**Figure 2:**
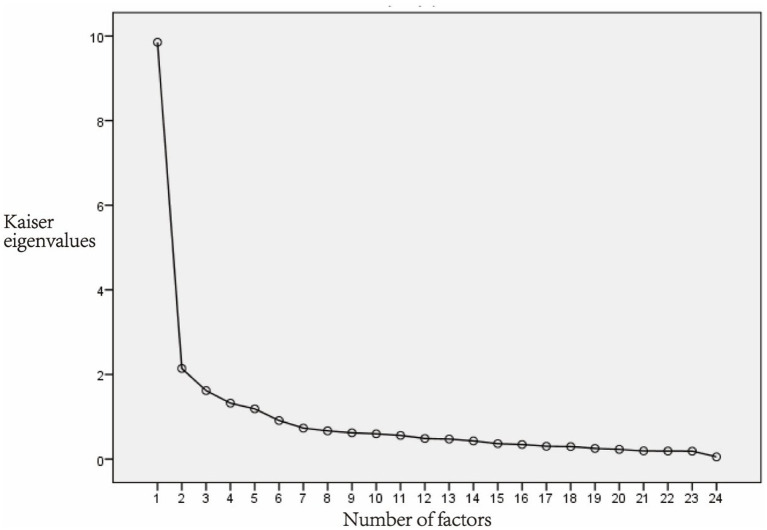
Scree plot.

All 25 items retained after item analysis were included in the EFA. The KMO of the 25-item scale was 0.916, the Chi-square of the Bartlett’s test of sphericity was 5734.828 (*p* < 0.001). The result indicated that the data were suitable for EFA. Principal component analysis and varimax rotation were used for analysis. The factors with eigenvalues >1 were extracted. After the first round EFA, 5 factors were extracted, and the cumulative explanatory variation was 66.030%. However, item15 had a factor loading of 0.368, which was <0.45 and was considered be deleted. Then the 24-items scale was tested for suitability of EFA. The KMO was 0.912, and the Chi-square of Bartlett’s test of sphericity was 5546.582 (*p* < 0.001), indicating that the data were suitable for EFA. There were five factors with eigenvalues >1. In the scree plot, the broken line became flat after the fifth factor (Figure 2). Finally, 5 factors were extracted, and the cumulative explanatory variation was 67.167%. The factor loading of each item on its corresponding factor was >0.45, ranging from 0.478 to 0.900 (Table 3). Factor 1 consists of 6 items named the Helplessness; Factor 2 consists of 5 items named Emotional; Factor 3 consists of 5 items named the Distress; Factor 4 consists of 5 items named Motivation; Factor 5 consists of 3 items named Guilt.

**Table 3:** The result of EFA (*N* = 356).

Item	Factor loading				
	1	2	3	4	5
18	0.791	0.238	0.054	0.128	0.196
26	0.779	0.272	0.235	0.164	0.243
27	0.713	0.417	0.160	0.183	0.156
24	0.679	0.079	0.322	0.037	0.302
29	0.635	0.431	0.138	0.245	0.148
17	0.580	0.079	0.381	0.143	0.249
9	0.118	0.752	0.112	0.238	0.032
5	0.202	0.749	0.177	0.048	0.169
7	0.185	0.702	0.313	0.099	0.161
8	0.235	0.665	0.172	0.121	0.223
6	0.393	0.520	0.419	0.093	0.176
28	0.239	0.224	0.772	0.130	0.069
25	0.202	0.219	0.687	0.171	−0.079
14	0.359	0.069	0.618	0.041	0.238
33	0.147	0.227	0.596	0.209	0.330
11	−0.008	0.167	0.572	0.252	0.047
2	0.004	0.092	0.159	0.840	0.144
3	0.050	0.149	0.226	0.774	0.135
1	0.172	0.168	0.051	0.764	−0.039
4	0.340	0.132	0.203	0.610	−0.018
13	0.400	−0.026	0.176	0.478	0.320
31	0.249	0.197	0.102	0.075	0.900
32	0.260	0.188	0.125	0.064	0.893
30	0.412	0.249	0.120	0.171	0.691
Eigenvalue	4.096	3.230	3.021	2.934	2.838
Variance explained by each factor (%)	17.068	13.459	12.588	12.227	11.825
Cumulative explanatory variation	17.068	30.527	43.116	55.342	67.167

Extraction method: Principal component analysis. Rotation method: Varimax with Kaiser normalization, rotate six times.

### 4.4. CFA

**Figure 3:**
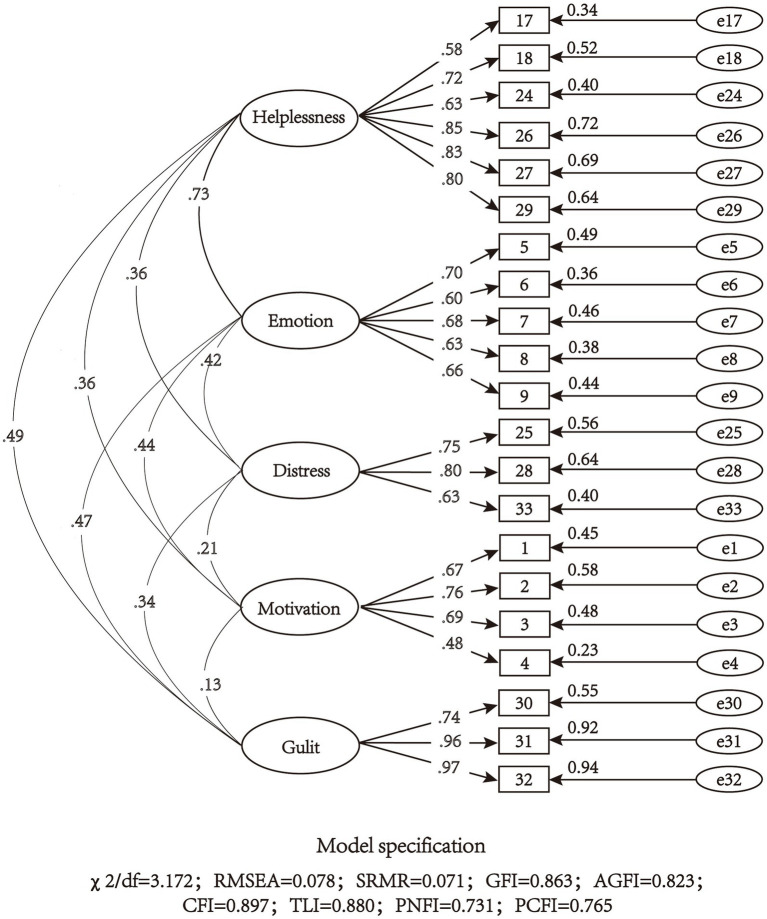
Model structure and standardized factor loadings of the five-factor model of the 21-item scale (*N* = 360).

The above 5 factors (24 items) scale was confirmed by CFA and Model 1 was obtained. The model fit indexed were *χ*^2^/df = 3.316, RMSEA = 0.080, SRMR = 0.092, GFI = 0.839, AGFI = 0.801, CFI = 0.860, TLI = 0.841, PNFI = 0.713, PCFI = 0.754, which indicated that the model fits poorly. In addition, CFA requires the factor loading of the items to be >0.40 (Nogales-González et al., 2015), so item 11 and 14 were removed. Item 13 was also removed for better model fitting though its factor loading is slightly higher than 0.40 (0.401). Then the 21-item scale was confirmed by Model 2. The *χ*^2^/df = 3.172, RMSEA = 0.078, SRMR = 0.071, GFI = 0.863, AGFI = 0.823, CFI = 0.897, TLI = 0.880, PNFI = 0.731, PCFI = 0.765 of model 2 were all within the acceptable range, which indicated that model 2 was acceptable (Figure 3).

### 4.5. Validity

The validity of the 5-factor 21-item scale generated by CFA was analyzed. PHQ-9 was used to test the criterion-related validity of the scale. The correlations between the five factors and PHQ-9 were 0.602, 0.490, 0.432, 0.489, and 0.604 (*p* < 0.05), respectively. The correlation coefficient between the total score and PHQ-9 was 0.731 (*p* < 0.05). All were in the range of 0.40–0.80.

Convergent validity was indicated by AVE (Table 4). The AVE for the five factors are 0.548, 0.427, 0.533, 0.432, and 0.804, respectively. The AVE of emotions and motivation did not reach 0.50, and the other factors reached the standard. Discriminant validity was expressed by comparing the square root of AVE with the correlation coefficients of a factor and other factors. In this study, the square root of AVE of each factor was larger than the correlation coefficient between that factor and the other factors, except that the square root of the AVE of factor 2 was smaller than its correlation coefficient with factor 1.

**Table 4:** Estimated correlations between domains and average variance extracted (AVE) of each domain (*N* = 360).

Domain	AVE	Factor 1	Factor 2	Factor 3	Factor 4	Factor 5
F1	**0.548**	**0.740**				
F2	**0.427**	0.727**	**0.653**			
F3	**0.533**	0.359**	0.417**	**0.730**		
F4	**0.432**	0.358**	0.441**	0.213*	**0.657**	
F5	**0.804**	0.492**	0.471**	0.336**	0.132*	**0.897**

**p < 0.001; *p < 0.05. The bold values mean the AVE and the square root of AVE of each factor.

### 4.6. Reliability

The Cronbach’s *α* of the total scale, helplessness, emotion, distress, motivation and guilt were0.892, 0.874, 0.779, 0.754, 0.727, and 0.914, which were >0.70. The ICC of the total scale and the five factors were 0.941, 0.901, 0.804, 0.904, 0.934, and 0.906, all of which were >0.75. The CR of each factor were 0.877, 0.788, 0.772, 0.747, and 0.897, all of which were >0.70.

## 5. Discussion

Through item analysis, EFA, and CFA, a 21-item PSDS-SS was developed. It includes 5 factors: helplessness (6 items), emotion (5 items), distress (3 items), motivation (4 factors) and guilt (3 items). The above three items reduction procedures are rigorous and have been used by many researchers (Jin et al., 2018; Li et al., 2019). The PSDS-SS has good reliability and validity, and is considered as an effective measurement to evaluate the sequelae stage PSD.

### 5.1. Factor structure of the scale

The PSDS-SS consists of 5 factors, which are helplessness, emotion, distress, motivation and guilt. Helplessness refers to a sense of helplessness caused by patients feeling that their current life and physical condition are out of control. The emotion factor included depressed mood, despair and other emotional changes of patients. Distress refers to the patients’ pessimistic view of social interactions and themselves. Motivation refers to patients’ willingness and initiative to engage in purposeful activities in daily life. Guilt means that patients feel indebted to their family because they bring great pressure to their family.

### 5.2. Revision of factors and items

The factors of cognitive, sleep, somatization and behavior in the original scale were deleted. The cognitive was removed after EFA. The sleep was removed after item analysis. About 80% of stroke patients experience cognitive decline and 38.2% of them experience insomnia (Umarova, 2017; Baylan et al., 2020; Iddagoda et al., 2020; Buvarp et al., 2021). Therefore, the inclusion of cognitive and sleep items in the scale may reduce the specificity of the scale in screening PSD in the sequelae stage. Somatization was removed from EFA. Items 17 and 18 of somatization were moved to the helplessness. Items 17 and 18 mainly express patients’ despair related to daily life and physical conditions that they could not change, so it is reasonable to move them to the helplessness. Behavior factor was removed from EFA. Specifically, item13 “I have fewer social activities” from the behavior factor was moved to the motivation factor. This item expresses the reduced initiative of patients to participate in social activities, so it is suitable to be placed in the motivation factor. Items 11, 14, 25, 28, and 33 of the cognitive, the somatization, the helplessness and guilt factors were incorporated into a new factor after the EFA. Since these 5 items mainly expressed the trouble of patients about the social environment and themselves, the factor was named “Distress.”

### 5.3. Validity and reliability of the PSDS-SS

Structural validity (EFA and CFA), criterion-related validity, convergent and discriminant validity were used to test the validity of the scale. (1) Structural validity: A total of 5 factors were extracted from EFA. The factor loadings of the 24 items reached the standard. For the CFA model with 5 factors and 21 items, all model fit indicators were within the acceptable range. The results of EFA and CFA indicate that the scale has good structural validity. (2) Criterion-related validity: The correlation between each factor and PHQ-9, and the total scale and PHQ-9 were all up to standard, indicating the criterion-related validity was good. (3) Convergent validity: Except for the emotion and motivation factors, the AVE of the other factors reached the standard. The AVE is calculated by the factor loadings. When calculating the AVE, the factor loading higher than 0.50 is acceptable (Liu et al., 2022). The factor loadings of all items in the emotion factor were >0.50, and the factor loadings of items in the motivation factor were >0.50 except for item 4 (which was close to 0.50). Thus, the convergent validity was also acceptable for the emotion and motivation factors. (4) Discriminant validity: Except for the discriminant validity of factors 2 and 1, all the other factors met the criterion of discriminant validity. Overall, the discriminant validity of this scale was acceptable (Sousa et al., 2022).

The Cronbach’ *α*, test–retest reliability and composite reliability (CR) were used to evaluate the reliability of the scale. The Cronbach’ *α* of each factor and total scale were >0.70, indicating good internal consistency of the scale. The ICC of all factors and total scale were >0.75, indicating that the scale has excellent test–retest reliability and has stability and consistency across time. The CR of all factors was >0.70, indicating good composite reliability.

## 6. Strengths and limitations

Compared with the current tools used to evaluate the sequelae stage PSD, the PSDS-SS is more practical and targeted. First, compared with the mental disease diagnostic systems, the PSDS-SS is convenient and can be used for patient self-assessment. Secondly, compared with the ordinary depression rating scale, this PSDS-SS was developed from the target population, so it is specific for measuring sequelae stage PSD with better sensitivity and specificity. Third, this PSDS-SS focuses more on the specific course of disease compared with the specific PSD screening scale. The items in the helplessness factor reflected the specific symptom of PSD in the sequelae stage. Specifically, they are the items 18, 24, 26, 27, 29 which came from interviews with PSD patients in the sequelae stage. During the interview, patients expressed a sense of helplessness arising from the long-term disease state, which differs from other scales. The items in the helplessness factor mentioned above manifested specific symptoms of PSD patients in the sequelae stage, which are dissatisfied with physical function, rehabilitation prognosis and life but unable to do anything about it. It can more accurately reflect the characteristics of PSD in a specific stage.

Several limitations should be noted for this study. First, the subjects were from only one hospital, which might limit the universality of the results; further multi-Centre study should be taken to confirm the suitability of the scale. Second, this is a cross-sectional study, longitudinal studies should be conducted to evaluate the dynamic effectiveness of this scale.

## 7. Conclusion

The PSDS-SS has acceptable reliability and validity that can be used to screen the depressive symptoms of stroke patients in the sequelae stage, which is helpful for the effective identification and symptom management of PSD in the sequelae stage.

## Data availability statement

The raw data supporting the conclusions of this article will be made available by the authors, without undue reservation.

## Ethics statement

The studies involving human participants were reviewed and approved by Research Review Board of the First Affiliated Hospital of Wenzhou Medical University. The patients/participants provided their written informed consent to participate in this study.

## Author contributions

YZ: research design and manuscript writing. FL, LN, and HL: data analysis. SL, BG, YG, and YF: data collection. JL: research design and manuscript revising. All authors contributed to the article and approved the submitted version.

## Funding

This work was supported by The Wenzhou Municipal Science and Technology Bureau (grant number: Y20220098); The Health Commission of Zhejiang Province (grant number: 2021KY780); and The Wenzhou Municipal Science and Technology Bureau (grant number: Y20220102).

## Conflict of interest

The authors declare that the research was conducted in the absence of any commercial or financial relationships that could be construed as a potential conflict of interest.

## Publisher’s note

All claims expressed in this article are solely those of the authors and do not necessarily represent those of their affiliated organizations, or those of the publisher, the editors and the reviewers. Any product that may be evaluated in this article, or claim that may be made by its manufacturer, is not guaranteed or endorsed by the publisher.
